# Socioeconomic Risk Factors for Incomplete Vaccination in Swedish Two‐Year‐Olds—A Nationwide, Population‐Based Cohort Study

**DOI:** 10.1111/apa.70582

**Published:** 2026-05-14

**Authors:** Cecilia Chrapkowska Almqvist, Ilias Galanis, Tiia Lepp, Malin Kark, Sharon Kühlmann‐Berenzon, Anna Nilsson

**Affiliations:** ^1^ Division of Paediatric Oncology, Dept of Women and Children's Health Karolinska Institutet Stockholm Sweden; ^2^ Unit for Immunisations Public Health Agency of Sweden Stockholm Sweden; ^3^ Unit for Advanced Paediatric Home Care Astrid Lindgren's Children's Hospital Stockholm Sweden; ^4^ Unit for Analysis Public Health Agency of Sweden Stockholm Sweden; ^5^ Unit for Public Health Systems Public Health Agency of Sweden Stockholm Sweden

**Keywords:** National Immunisation Programme, preventive health services, social determinants of health, vaccination coverage

## Abstract

**Aim:**

To analyse the association between specific socioeconomic measures and the risk of incomplete vaccination for diphtheria‐tetanus‐pertussis (DTP) and measles‐containing vaccine (MCV) in Swedish two‐year‐olds.

**Methods:**

This nationwide, population‐based cohort study included 227 457 children born in Sweden during 2014–2015. Data were obtained from the National Vaccination Register (NVR) and linked to socioeconomic data from Statistics Sweden. Multivariable logistic regression was used to estimate the associations between family characteristics—including income, education, employment, and family structure—and incomplete DTP and MCV vaccination at 24 months of age.

**Results:**

Incomplete vaccination was found in 13.1% of children for DTP3 (three doses) and 8.7% for MCV1 (one dose). Low family income, single parenthood, parental unemployment, frequent residential mobility, and parental immigration were risk factors for incomplete vaccination. A previously unreported risk factor was having a sibling with a disability or chronic disease, which increased the risk of incomplete DTP3 (aOR 1.15, 95% CI 1.05–1.27) and MCV1 (aOR 1.27, 95% CI 1.14–1.41).

**Conclusion:**

Despite a tax‐funded national immunisation programme, socioeconomic disparities in vaccination coverage exist in Sweden. Children in socially vulnerable families, and those with siblings requiring disability support, need targeted interventions to ensure full immunisation.

## Summary

- Socioeconomic disparities in childhood vaccination coverage exist even in high‐income countries with free National Immunisation Programmes, though findings have been inconsistent.
- This nationwide, population‐based Swedish cohort study identifies low income, single parenthood, unemployment, residential mobility, and—uniquely—having a sibling with a chronic disease as risk factors for incomplete vaccination.
- Targeted policies and supportive structures are necessary to ensure equitable access and maintain high vaccination coverage among these vulnerable families.

**Abbreviations**
aOR: adjusted Odds Ratio
COVID‐19: Coronavirus Disease 2019
DTP: Diphtheria‐Tetanus‐Pertussis
HPV: Human Papilloma Virus
LISA: Longitudinal Integrated Database for Health Insurance and Labour Market Studies
MCV: Measles‐Containing Vaccine
NIPs: National Immunisation Programmes
NVR: National Vaccination Register
Q: Quintiles
RR: Relative Risk
RTB: Register of the Total Population
SEK: Swedish currency
VPD: Vaccine Preventable Disease
WHO: World Health Organisation

## 1 Introduction

Childhood vaccination has contributed to major advancements in health, saved many lives and led to the eradication of smallpox in 1980 [1]. Today, more than 80% of two‐year‐olds globally have received vaccines against at least six diseases through National Immunisation Programmes (NIPs) [2]. A high and even coverage for vaccinations included in NIPs protects vaccinated individuals by the development of active immunity, and the entire population through herd immunity. With the re‐emergence of vaccine‐preventable diseases (VPDs), reducing inequalities in vaccination coverage in both high‐ and low‐income countries is a prioritised public health goal by the WHO and the European Council [3].

Factors influencing vaccine uptake include socioeconomic inequalities, which may impact vaccine hesitancy and barriers to use the immunisation services [4, 5]. Socioeconomic status is defined as a composite of educational level, income, and occupation, and is measured as one of these or a combination of these, sometimes merged into different indices. Unemployment rates are occasionally also included [6]. For childhood vaccination outcomes, most earlier studies have used area‐based measures of socioeconomic position. Studies with individual‐level data are less common, and data on both income and education on the same subjects are seldom reported [7, 8]. Systematic reviews on socioeconomic position and childhood vaccination in high‐income countries have emphasised the inconsistency of results, where some studies report lower and some higher rates of vaccination for children with low socioeconomic position, and others report no significant effects [5, 9]. However, for low‐ and middle‐income countries, children in families with low income and mothers without any formal education are more consistently at risk of being incompletely immunised [10]. We were also interested in exploring whether family‐related factors such as immigration, parental sick leave, disability in siblings or being a single parent could impact vaccination status [11, 12].

In Sweden, the NIP offers vaccinations free of charge to all resident children and is delivered by nurse‐led well‐baby clinics. Vaccine containing diphtheria, tetanus, and pertussis antigens (DTP‐containing vaccine) is scheduled at 3, 5, and 12 months of age, and measles‐containing vaccine (MCV) at 18 months of age. Since 2013 all NIP vaccinations administered must be recorded in the National Vaccination Registry (NVR). Before 2013 the national vaccination coverage was monitored by an administrative system [13].

Historically, Sweden has had a high and stable vaccination coverage for DTP‐ and MCV‐containing vaccines. This suggests a high parental confidence in the NIP organised and provided by the Public Health Agency, regional health care systems and the well‐baby clinics [14, 15]. The objective of this study was to analyse the association between specific socioeconomic measures and the risk of incomplete vaccination status of DTP3 and MCV1 at two years of age in a Swedish total‐population birth cohort.

## 2 Patients and Methods

### 2.1 Population

The birth cohort included all children born in Sweden from January 1st 2014, to December 31st 2015, with a Swedish personal identity number. The study cohort was identified from the Register of the Total Population (RTB) at Statistics Sweden as described by Chrapkowska et al. [13]. Death and migration dates of the children were obtained from the RTB; birth dates from the NVR or the RTB.

### 2.2 Outcome Variables

Each of the two outcomes, incomplete vaccination with DTP3 and incomplete vaccination with MCV1, was analysed separately based on the WHO standard indicators for immunisation coverage [8]. For DTP, incomplete vaccination was defined as having received less than 3 doses of DTP‐containing vaccine by 24 months of age. Since the 3rd dose is scheduled at 12 months of age, we allowed for 12 additional months for completing the recommended number of DTP vaccine doses.

For MCV, incomplete vaccination was defined as not having received a dose at 24 months of age. The 1st dose is scheduled at 18 months; thus, we allowed an additional six months before classifying the vaccination as incomplete.

### 2.3 Explanatory Variables

The following socioeconomic indicators recommended by the WHO were included [10, 16]: child's sex, birth order, maternal age, parental education, and household economic status. Variables that reflected the social or family situation of the child were added: unemployment of one or both parents, sick leave of one or both parents, single parent, sibling with disability support, parental immigration to Sweden before the birth of the index child, and residential mobility within Sweden. Values for the social or family situation were parental values from the birth year of each child from the Longitudinal Integrated Database for Health Insurance and Labour Market Studies (LISA) database. Original and our variable names are specified in the Table S2. The definitions and sources for the original variables are available from Statistics Sweden.

*Maternal age* was categorised as follows: < 25, 25–29, 30–34 and ≥ 35 years of age, birth order into 1, 2, 3, and≥ 4; *parental education* into low < 10 years, medium 10–12 years and high > 12 years; and *residential mobility* into 0, 1 or ≥ 2 relocations during calendar year of the child's birth.

For *unemployment*, the categories were 0, 1, or 2 parents unemployed during the child's birth year. If the information was not available for one of the parents, the variable was set to missing.

For *education* and *residential mobility*, the highest level of education and, respectively, the highest number of relocations between the two parents, was set as the family value. When the information was missing for one of the parents, the information of the other parent was used. For the variables *parental sick leave* and *sibling with disability*, the family value was defined as follows: As positive if at least one parent had received support for sick leave or a sibling with disability. If one parent had not received support for sick leave or a sibling with disability, and the information for the other parent was missing, it was defined as “missing”. For all variables, if a value was missing for both parents, the family variable was set to “missing”.

*Family income* was measured as equivalised disposable income, calculated by Statistics Sweden for all households based on data from the national taxation and the population register. For children whose two parents belonged to different households, the mean income for the two households was set as family income. When the information was missing for one of the parents, the income of the other parent was used. In cases where income was either zero or missing for both parents, the income was set to “missing”. For 679 children, at least one parent had a negative disposable income. It was assumed that parents with negative income differed from those with low but positive income, and we decided to set their income to “missing” [17].

The mean annual family income was grouped into quintiles, Q1–Q5, where Q1 is the lowest. The upper limit for Q1 was 127 599 SEK, while the lower limit for Q5 was 259 500 SEK. The limits for low economic standard (< 60% of the median disposable income in the entire population) were 151 380 SEK in 2014 and 156 840 SEK in 2015 [17], meaning that all children in Q1 and some in Q2 had a low economic standard.

### 2.4 Statistical Methods

The child's birthdate was considered the cohort entry date. Children who were lost to follow‐up, either due to emigration or death within two years of life, were excluded from the analysis.

Univariate logistic regressions were first fitted to estimate the impact on the outcomes of maternal and paternal values of education, parental sick leave, and sibling with disability support separately. Maternal and paternal values influenced the outcomes similarly, so we created family variables for each of these exposures.

We fitted logistic regression to each outcome using complete cases. Univariable logistic regression was used to assess each explanatory variable, and those with a *p*‐value < 0.2 were included in a multivariable model.

For DTP3, the variable “Child's sex” was the only variable initially excluded from the multivariable model. Backwards selection using the likelihood ratio test with *p*‐value < 0.05 was then performed, leading to the exclusion of the variable “Parental sick leave”. Exclusion of this variable had a limited effect on the estimates of the other variables (< 10%). The likelihood ratio test didn't suggest any further exclusions. The variable “Child's sex” was then included in this model, but it was not significant based on the likelihood ratio test, and, similar to above, including it in the model had a limited effect on the estimates of the other variables. Hence, the variable “Child's sex” was also excluded from the final model.

For MCV1, a similar process was applied. The variables initially excluded from the multivariable analysis were “Child's sex” and “Parental sick leave”. Based on the likelihood ratio test, all variables in the multivariable model were significant. The variables “Child's sex” and “Sick leave” were then added one by one to the model. “Child's sex” was not significant and had a limited effect on the estimates of the other variables. On the other hand, “Parental sick leave” was now significant based on the likelihood ratio test and was added to the final model for MCV1. The variable “Child's sex” was hence the only variable excluded from the final model.

Results are reported as crude and adjusted odds ratios with 95% confidence intervals. The generalised variance inflation factor [18] was used to evaluate multicollinearity between the covariates and the Le Cessie‐van Houwelingen‐Copas‐Hosmer test to assess the goodness‐of‐fit of the final models [19]. The statistical software R (https://www.r‐project.org/) was used for analyses, and *p*‐values < 0.05 were considered statistically significant.

## 3 Results

### 3.1 Vaccination Status in the Cohort

The cohort consisted originally of 229 750 children. After excluding 2 181 children due to emigration, 111 children due to death before 2 years of age and one child due to a missing birth date, 227 457 (99.0%) remained in the study cohort. The number of individuals with at least one missing value for explanatory variables, leading to their exclusion from the multivariable logistic regression, was 8725 (3.8%).

Table 1 shows the characteristics of the included infants. Most children, 199 346 (87.6%), lived with cohabiting parents, and 136 391 (60.0%) had at least one parent with higher education. Only 13 090 (5.8%) had no parent with at least 10 years of education.

**TABLE 1:** Cohort characteristics.

	Number	Proportion
Total	227 457	100%
Child's sex		
Female	110 082	48.4%
Male	117 375	51.6%
Missing	0	
**Maternal age**		
< 25	25 856	11.4%
25–29	68 181	30.0%
30–34	77 759	34.2%
≥ 35	55 463	24.4%
Missing	198	0.1%
**Family type**		
Two‐parent household	199 346	87.6%
One‐parent household	27 680	12.2%
Missing	431	0.2%
**Birth order**		
First	96 819	42.6%
Second	85 495	37.6%
Third	31 651	13.9%
≥ Fourth	13 138	5.8%
Missing	354	0.2%
**Parental education**		
High > 12 years	136 391	60.0%
Medium 10–12 years	76 818	33.8%
Low < 10 years	13 090	5.8%
Missing	1 158	0.5%
**Unemployment**		
No	157 902	69.4%
One parent	46 712	20.5%
Two parents	16 017	7.0%
Missing	6 826	3.0%
**Parental sick leave**		
No	149 350	65.7%
Yes	72 408	31.8%
Missing	5 699	2.5%
**Sibling with disability**		
No	217 158	95.5%
Yes	3 568	1.6%
Missing	6 731	3.0%
**Residential mobility**		
No relocations	161 850	71.2%
One relocation	55 199	24.3%
≥ Two relocations	10 385	4.6%
Missing	23	0.0%
**Parental immigration**		
No	150 508	66.2%
One parent	35 086	15.4%
Both parents	41 863	18.4%
Missing	0	
**Family income (SEK/year)**		
Q5 ≥ 259 500	45 259	19.9%
Q4 208 000–259 499	45 155	19.9%
Q3 171 500–207 999	45 358	19.9%
Q2 127 600–171 499	45 240	19.9%
Q1 1–127 599	45 284	19.9%
Missing	1 161	0.5%

A total of 29 720 (13.1%) children had incomplete DTP3 at 24 months of age. Incomplete MCV1 at the same age was detected in 19 737 (8.7%) children (Table 2). Among the children with incomplete DTP3, 8 466 (28.5%) had incomplete MCV1 compared to 11 271 (5.7%) of children with complete DTP3 having incomplete MCV1. Thus, the risk of having incomplete MCV1 was 5.0 (95% CI 4.9–5.1) times higher for a child with incomplete DTP3 compared to a child with complete DTP3.

**TABLE 2:** Incomplete and complete vaccination with DTP3 and MCV1 in Swedish two‐year‐olds.

	Complete MCV1		Incomplete MCV1		Sum	
	*N*	%^a^	*N*	%^a^	*N*	%^a^
Complete DTP3	186 466	82.0%	11 271	5.0%	197 737	86.9%
Incomplete DTP3	21 254	9.3%	8 466	3.7%	29 720	13.1%
Sum	207 720	91.3%	19 737	8.7%	227 457	100%

^a^ Percentages are per cent of the total population.

### 3.2 Variables Affecting the Risk for Incomplete DTP3 and MCV1

Table 3 shows the associations between explanatory variables and risk of incomplete DTP3 status, and Table 4 shows the same for MCV1. Most of the studied socioeconomic measures were risk factors for incomplete vaccination for both DTP3 and MCV1 among children born in 2014–2015.

**TABLE 3:** Incomplete vaccination with three doses of DTP–containing vaccine (DTP3) at 24 months of age.

	Incomplete DTP3 (*n*)	Incomplete DTP3 (%)	Crude odds ratio (95% CI)	Adjusted odds ratio (95% CI)
Total	29 720	13.1		
**Child's sex**				
Female	14 337	13.0	Ref.	
Male	15 383	13.1	1.01 (0.98–1.03)	—
Missing	0			
**Maternal age**				
< 25	3 643	14.1	1.12 (1.07–1.16)	0.95 (0.90–0.99)
25–29	8 613	12.6	0.99 (0.96–1.02)	0.95 (0.92–0.98)
30–34	9 952	12.8	Ref.	Ref.
≥ 35	7 410	13.4	1.05 (1.02–1.09)	1.06 (1.02–1.09)
Missing	102	51.5		
**Family type**				
Two‐parent household	24 864	12.4	Ref.	Ref.
One‐parent household	4 779	17.3	1.46 (1.42–1.51)	1.14 (1.10–1.19)
Missing	77	17.9		
**Birth order**				
First	13 050	13.5	Ref.	Ref.
Second	10 756	12.6	0.92 (0.90–0.95)	0.95 (0.92–0.97)
Third	4 033	12.7	0.94 (0.90–0.97)	0.92 (0.89–0.96)
≥ Fourth	1 817	13.8	1.03 (0.98–1.09)	0.93 (0.87–0.99)
Missing	64	18.1		
**Parental education**				
> 12 years	17 403	12.8	Ref.	Ref.
10–12 years	9 979	13.0	1.02 (0.99–1.05)	0.97 (0.95–1.00)
< 10 years	2 057	15.7	1.27 (1.21–1.34)	0.93 (0.87–0.99)
Missing	281	24.3		
**Parental unemployment**				
No	18 751	11.9	Ref.	Ref.
One parent	6 635	14.2	1.23 (1.19–1.27)	1.16 (1.12–1.20)
Two parents	2 712	16.9	1.51 (1.45–1.58)	1.35 (1.27–1.44)
Missing	1 622	23.8		
**Parental sick leave**				
No	19 193	12.9	Ref.	—
Yes	9 126	12.6	0,98 (0,95–1 × 00)	—
Missing	1 401	24.6		
**Sibling with disability**				
No	27 585	12.7	Ref.	Ref.
Yes	528	14.8	1,19 (1,09–1 × 31)	1.15 (1.05–1.27)
Missing	1 607	23.9		
**Residential mobility**				
No relocations	20 488	12.7	Ref.	Ref.
One relocation	7 595	13.8	1,10 (1,07–1,13)	1.07 (1.04–1.10)
≥ Two relocations	1 624	15.6	1.28 (1.21–1.35)	1.14 (1.08–1.21)
Missing	13	56.5		
**Parental Immigration**				
No parent	18 573	12.3	Ref.	Ref.
One parent	5 334	15.2	1.27 (1.23–1.32)	1.11 (1.07–1.15)
Both parents	5 813	13.9	1.15 (1.11–1.18)	0.97 (0.93–1.00)
Missing	0			
**Family income (SEK/year)**				
Q1 1–127 599	7 224	16.0	1.37 (1.32–1.43)	1.14 (1.08–1.21)
Q2 127 600–171 499	5 866	13.0	1.08 (1.04–1.12)	1.06 (1.01–1.11)
Q3 171 500–207 999	5 512	12.2	1.00 (0.96–1.04)	1.03 (0.98–1.07)
Q4 208 000–259 499	5 290	11.7	0.96 (0.92–1.00)	0.98 (0.94–1.02)
Q5 ≥ 259 500	5 490	12.1	Ref.	Ref.
Missing	1 161	29.1		

*Note:* The variables “Child's sex” and “Parental sick leave” are not included in the final multivariable model.

**TABLE 4:** Incomplete vaccination with one dose of measles–containing vaccine (MCV1) at 24 months of age, 2014–2015.

	Incomplete vaccination (*n*)	Incomplete vaccination (%)	Crude odds ratio (95% CI)	Adjusted odds ratio (95% CI)
Total	19 737	8.7		
**Child's sex**				
Female	9 480	8.6	Ref.	—
Male	10 257	8.7	1.02 (0.99–1.05)	—
Missing	0			
**Maternal age**				
< 25	2 588	10.0	1.25 (1.19–1.31)	1.06 (1.00–1.13)
25–29	5 839	8.6	1.05 (1.01–1.09)	1.02 (0.98–1.06)
30–34	6 352	8.2	Ref.	Ref.
≥ 35	4 907	8.8	1.09 (1.05–1.13)	1.06 (1.01–1.10)
Missing	51	25.8		
**Family type**				
Two‐parent household	16 074	8.1	Ref.	
One‐parent household	3 611	13.0	1.71 (1.65–1.78)	1.38 (1.32–1.45)
Missing	52	12.1		
**Birth order**				
First	8 059	8.3	Ref.	Ref.
Second	7 188	8.4	1.01 (0.98–1.05)	1.04 (1.00–1.07)
Third	2 920	9.2	1.12 (1.07–1.17)	1.09 (1.04–1.15)
≥ Fourth	1 524	11.6	1.45 (1.36–1.53)	1.23 (1.15–1.31)
Missing	46	13.0		
**Parental education**				
> 12 years	10 966	8.0	Ref.	Ref.
10–12 years	7 027	9.1	1.15 (1.12–1.19)	1.02 (0.99–1.06)
< 10 years	1 549	11.8	1.54 (1.45–1.62)	0.95 (0.88–1.02)
Missing	195	16.8		
**Parental unemployment**				
No	11 906	7.5	Ref.	Ref.
One parent	4 761	10.2	1.39 (1.34–1.44)	1.15 (1.10–1.21)
Two parents	1 967	12.3	1.72 (1.63–1.81)	1.24 (1.15–1.33)
Missing	1 103	16.2		
**Parental sick leave**				
No	12 657	8.5	Ref.	
Yes	6 131	8.5	1.00 (0.97–1.03)	1.06 (1.03–1.10)
Missing	949	16.7		
**Sibling with disability**				
No	18 222	8.4	Ref.	Ref.
Yes	428	12.0	1.49 (1.34–1.65)	1.27 (1.14–1.41)
Missing	1 087	16.1		
**Residential mobility**				
No relocations	13 607	8.4	Ref.	Ref.
One relocation	4 987	9.0	1.08 (1.05–1.12)	1.03 (0.99–1.06)
≥ Two relocations	1 133	10.9	1.33 (1.25–1.42)	1.09 (1.02–1.17)
Missing	10	43.5		
**Parental Immigration**				
No parent	11 884	7.9	Ref.	Ref.
One parent	3 608	10.3	1.34 (1.29–1.39)	1.16 (1.11–1.21)
Both parents	4 245	10.1	1.32 (1.27–1.37)	1.05 (1.01–1.10)
Missing	0			
**Family income (SEK/year)**				
Q1 (1–127 599)	5 468	12.1	1.75 (1.68–1.84)	1.30 (1.22–1.39)
Q2 (127 600 – 171 499)	4 125	9.1	1.28 (1.22–1.34)	1.13 (1.07–1.19)
Q3 (171 500 – 207 999)	3 480	7.7	1.06 (1.01–1.12)	1.01 (0.96–1.07)
Q4 (208 000 – 259 499)	3 189	7.1	0.97 (0.92–1.02)	0.96 (0.91–1.01)
Q5 (≥ 259 500)	3 285	7.3	Ref.	Ref.
Missing	190	16.4		

*Note:* The variable “Child's sex” is not included in the final multivariable model.

Maternal age affected incomplete vaccination in both DTP3 and MCV1. Children born to mothers aged > 35 years were more likely to be incompletely vaccinated than children born to mothers 30–34 years of age (aOR 1.06, 95% CI 1.02–1.09) for DTP. On the other hand, younger maternal age was associated with less incomplete vaccination for DTP3. For MCV1, children born to both mothers < 25 years and mothers aged > 35 years were more likely to be incompletely vaccinated.

In families with a single parent, the aOR for incomplete DTP3 was 1.14 (95% CI 1.10–1.19) compared to families with cohabiting parents, while the association for incomplete MCV1 was even higher with aOR 1.38 (95% CI 1.32–1.45) in households with one parent. Parental education affected DTP3, where children born to parents with < 10 years of education were less likely to be incompletely immunised (aOR 0.93, 95% CI 0.87–0.99) while there was no impact on MCV1 (Table 3 and Table 4).

Family income was associated with incomplete vaccination for both DTP3 and MCV1, where children in families with the lowest income quintiles (Q1 and Q2) were more likely to be incompletely vaccinated. In addition, children with two unemployed parents were more likely to have incomplete DTP3 (aOR 1.35, 95% CI 1.27–1.44) compared to children without unemployed parents. This was also noticed for incomplete MCV1 (aOR 1.24, 95% CI 1.15–1.33). Parental short‐term sick leave did not affect vaccination for DTP3, but it was associated with incomplete vaccination for MCV1 (aOR 1.06, 95% CI 1.03–1.10). However, in families where a parent received support for a sibling with a disability or chronic disease, children more often had incomplete DTP3 (aOR 1.15, 95% CI 1.05–1.27) as well as incomplete MCV1 (aOR 1.27, 95% CI 1.14–1.41) compared to children living without siblings with disability/chronic disease.

Since well‐baby clinics traditionally have been responsible for providing NIP to children residing in the neighbouring area, we investigated whether relocations during the child's first year affected incomplete vaccination. Interestingly, children whose parents had relocated more than twice during their first year of life had incomplete DTP3 (aOR 1.14, 95% CI 1.08–1.21) as well as incomplete MCV1 (aOR 1.09, 95% CI 1.02–1.17) compared to children without relocations. Parental immigration also affected incomplete vaccination; children with one parent born abroad had incomplete DTP3 (aOR 1.11, 95% CI 1.07–1.15) compared to children with parents born in Sweden. Incomplete MCV1 was also more common in children where one or both parents had immigrated to Sweden (Table 4).

The higher the birth order, the higher the likelihood of incomplete MCV1 (aOR 1.23, 95% CI 1.15–1.31) for a child with ≥ 3 older siblings compared to a first‐born child. Contrarily, a higher birth order was associated with a lower likelihood of incomplete DTP3 (aOR 0.93, 95% CI 0.87–0.99) for the same comparison.

## 4 Discussion

To the best of our knowledge, this is the largest report to date studying the impact of socioeconomic and family factors on vaccination coverage in preschool children, as well as the first total‐population cohort study with individual‐level data on family income, education, and family characteristics. In addition, this is the first study to report an increased risk for incomplete vaccination among children having a sibling with a disability or chronic disease.

In a high‐income country, we show that indicators of socioeconomic position, such as single parenthood, low income and unemployment, are associated with an increased risk of incomplete immunisation for both DTP3 and MCV1 in children at two years of age. Children incompletely vaccinated with one vaccine had a high risk of incomplete vaccination with the other, as shown in the UKMillennium Study Cohort. In the UK cohort consisting of 14 578 children born in 2000–2002, the relative risk (RR) for being unimmunised with MCV at three years of age was 6.0 (95% CI 5.1 to 7.1) for those partially immunised with DTP3, which is similar to our RR of 5.0 (95% CI 4.9–5.1) [20].

In agreement with earlier studies from Europe [7, 9, 21, 22], North America [23] and Africa [6] children living with a single parent were at increased risk for incomplete vaccination for DTP3 as well as MCV1. As a single parent, it may be more difficult to allocate time for vaccination at the well‐baby clinic as five visits are scheduled for immunisations during the child's first two years. It might also be possible that travelling to a well‐baby clinic could be a more important barrier for single parents, even though well‐baby clinics are based on units in residential areas. Other known barriers to vaccination, such as direct costs or parental consent to vaccination, are less likely to explain the differences between families with one or two parents, as the system is free of charge and oral consent from parents is sufficient for vaccination [15]. Our data also suggests that the risk of incomplete MCV1 is higher than for DTP3, which may be related to the structure of parental leave in Sweden, where many parents have returned to work after the child's first year. The finding of a higher risk of incomplete MCV1 in children of a higher birth order may also indicate that families with > 3 children struggle to find time for the visit to the well‐baby clinic and therefore miss out on MCV1. The association between a high birth order and incomplete MCV1 status is also in agreement with earlier results from Europe, Asia, South and North America, and Africa [6, 24]. Having a sibling with a disability or chronic disease was also a risk factor for incomplete DTP3 and MCV1, which we are the first to report. We may only speculate that this is an additional burden to families where time constraints may impact vaccination readiness.

Not surprisingly, lower income was associated with a higher risk of incomplete DTP3 and MCV1 vaccination in the present study, a result in line with previous findings from Europe [9], as well as from the 2008 birth cohort of 41 515 children from the Canadian province of Alberta [23]. Data from low‐ and medium‐income countries (reviewed in [12]) consistently show that children in the poorest wealth quantile are less likely to be vaccinated with DTP3 when comparing Q1 to Q5. Our result showing that incomplete MCV1 is more commonly found in Q1 and Q2 aligns with earlier studies from Sweden. In a regional birth cohort of 35 767 children, incomplete MCV1 in Q1 was more commonly found than in Q5 [14]. In a cohort of 25 000 full‐term children born in Sweden between 1998 and 2006, 6.1% had incomplete MCV1 in the highest income quartile and 9.5% in the lowest [25]. Unfortunately, no multivariable results are available for vaccination outcomes in these publications. Also, the finding of increased risk for incomplete vaccination in children with unemployed parents corroborates findings from Canada [26] and the UK [27].

The finding that residential mobility was associated with incomplete vaccination adds to previous studies from the UK [20] and Canada [23]. In the UK Millennium cohort, residential mobility was associated with increased risk of incomplete vaccination with DTP3 and MCV1. However, it was not associated with not being immunised at all against DTP or choosing a single‐antigen MCV [20]. This pattern could be better explained by drop‐out from the programme than by parental hesitancy or refusal of vaccinations. That may also be the case in the present study, especially among children in families moving more than once during their first year of life. In our setting, parents must contact a new local well‐baby clinic to book an appointment for vaccination after a relocation. From that perspective, it is encouraging that well‐baby clinics that offer extended home visit programmes also show an increased MCV vaccination rate compared to clinics without this programme [4].

Parental migration history was associated with a slightly increased risk of incomplete vaccination in the present study. Earlier studies from Italy and Australia covering children born 1995–2012 have shown a mixed pattern, where immigrant parents still have a higher risk of incomplete vaccination than non‐immigrant parents. However, in Italy, it was also noted that highly educated non‐immigrant mothers had begun to refuse vaccination for their children [7, 28]. It has also been shown in Sweden that some immigrant parents from eastern Africa are less likely to accept MCV for their children before the age of 24 months [29]. Probably, the dichotomous variable of migrant/non‐migrant background is too simplified to capture the full influence that migration might have on vaccination outcomes.

Parental education did not have a major effect on vaccination in our cohort; albeit it slightly decreased the risk of incomplete DTP3 in children born to parents with less than 10 years of education, while there was no impact for higher education. On the other hand, there are examples of settings where higher education is associated with a higher risk of incomplete vaccination for MCV1, such as the UK Millennium cohort [27] and a northern Italian 2005–2010 but not 1995–2005 cohort [30].

Our study has some strengths and limitations. The inability to opt out of the population or the vaccination registers minimised the risk of inclusion bias. An important strength of the study lies in the fact that the exposures and outcomes were recorded separately, meaning that our findings are unaffected by any recall bias. However, children without a Swedish personal number and children immigrating to Sweden during their first two years of life were not included in this study. Therefore, we do not capture the situation for the most vulnerable children of undocumented parents, who are known to avoid presenting to healthcare services [10].

Risk of classification bias for exposure variables was minor, due to the well‐characterised variables originating from state registers of economic benefits or tax reports. Some misclassification of the outcome is likely due to vaccines administered but not reported to the NVR [13]. As it is unlikely for this underreporting to differ by exposure status, this misclassification is expected to be non‐differential. We acknowledge that such misclassification may bias effect estimates, and our results should be interpreted with this limitation in mind, as we cannot fully exclude that low reporting rates to the NVR may co‐vary with well‐baby clinics with many socially disadvantaged families. It should also be noted that the follow‐up period for DTP 3 was significantly longer than for MCV1.

**To summarise**, addressing inequities in immunisation within high‐income countries requires targeted policies and supportive structures to ensure access for disadvantaged groups [3]. Our study demonstrates that disparities exist in the outcomes of the Swedish NIP. Specifically, we found that low family income, single parenthood, and parental unemployment are associated with an increased risk of children being incompletely immunised—findings that align with previous research on COVID‐19 [31] and HPV vaccination uptake among school‐aged girls in Sweden [32]. Additionally, our study identifies a previously not formally recognised at‐risk group: Children living with a sibling who has a chronic disease or disability are also at greater risk of incomplete vaccination.

## Author Contributions

**Anna Nilsson:** conceptualization, funding acquisition, writing – review and editing, supervision, resources, formal analysis, writing – original draft, validation. **Cecilia Chrapkowska Almqvist:** conceptualization, funding acquisition, writing – original draft, writing – review and editing, methodology, validation, formal analysis, project administration, data curation, investigation. **Sharon Kühlmann‐Berenzon:** writing – review and editing, formal analysis. **Tiia Lepp:** writing – review and editing. **Ilias Galanis:** investigation, writing – review and editing, validation, methodology, software, formal analysis, data curation. **Malin Kark:** conceptualization, investigation, writing – review and editing, validation, supervision, formal analysis, methodology.

## Funding

This work was supported by Region Stockholm, Stiftelsen Sven Jerrings Fond, Stiftelsen Samariten, and Stiftelsen Barnforskningen vid Astrid Lindgrens Barnsjukhus.

## Ethics Statement

The Study Was Conducted According to the Guidelines of the Declaration of Helsinki and Approved by the Swedish Ethical Review Authority (D‐Nr 2015/1961‐31‐4; 2016–1131‐32; 2017/2006–32).

## Consent

Patient consent was waived as only registry data was used in this study.

## Conflicts of Interest

The authors declare no conflicts of interest.

## Supporting information


**TABLE S1:** Vaccine names and categories.
**Table S2:** Exposure variables.

## Data Availability

The data supporting the findings of this study is available from Statistics Sweden and the Public Health Agency of Sweden, subject to ethical review as required by the Ethical Review Act (2003:460) and the Public Access to Information and Secrecy Act (2009:400), as well as other applicable legal requirements. Access to these data is restricted and was granted under license for the current study.
