# Supplementary material for: Socioeconomic Risk Factors for Incomplete Vaccination in Swedish Two‐Year‐Olds—A Nationwide, Population‐Based Cohort Study
Source: Acta Paediatr. 2026 May 14;115(11):2555–65. doi: 10.1111/apa.70582 (PMC13659413; doi:10.1111/apa.70582)
Supplement: Supplementary file 1 [file APA-115-2555-s001.docx]

# Supplementary data Socioeconomic risk factors for incomplete vaccination in Swedish two-year-olds – a population-based cohort study – variable definitions and categorization

**Supplementary table 1. Vaccine names and categories**

| **Vaccine category** | **Product names** |
| --- | --- |
| **DTP-containing** | Hexyon, Infanrix hexa, Infanrix hexa without Hib, Infanrix, Infanrix polio + Hib,  Infanrix Polio, Pentavac, Pentavac without Hib, Tetravac, Tetraxim, Tetravac  Acellulaire, Pentaxim |
|  |  |
| **MMR** | M-M-RVAXPRO, Priorix |

**Supplementary table 2. Exposure variables**

| **Variable name in article** | **Original Register** | **Variable name in Register** | **Time for data capture** |
| --- | --- | --- | --- |
|  |  |  |  |
| **Child’s sex** | **RTB** | Kon | The 31^st^ December of birth year |
| **Maternal age** | **RTB** | FoddArMor | The 31^st^ December of birth year |
| **Family type** | **RTB** | FamTypF | The 31^st^ December of birth year |
| **Birth order** | **RTB** | AntalBarnUnder18 | The 31^st^ December of birth year |
| **Parental education** | **LISA** | Sun2000niva | Birth year of the child |
| **Unemployment** | **LISA** | SyssStat11 | Birth year of the child |
| **Parental sick leave** | **LISA** | SjukP_Bdag | Birth year of the child |
| **Sibling with disability** | **LISA** | VardBidr | Birth year of the child |
| **Residential mobility** | **LISA** | AntFlyttTot | Birth year of the child |
| **Parental immigration** | **RTB** | UtlSvBakg | Birth year of the child |
| **Family income** | **LISA** | DispInkKE04 | Birth year of the child |
